# Supplementary material for: The ER calcium channel Csg2 integrates sphingolipid metabolism with autophagy
Source: Nat Commun. 2023 Jun 22;14:3725. doi: 10.1038/s41467-023-39482-6 (PMC10287731; doi:10.1038/s41467-023-39482-6)
Supplement: Supplementary file 1 — Supplementary Information [file 41467_2023_39482_MOESM1_ESM.pdf]

Supplementary Information for

The ER Calcium Channel Csg2 Integrates Sphingolipid Metabolism with Autophagy

Shiyan Liu[1,#], Mutian Chen[2,3,#], Yichang Wang[4,#], Yuqing Lei[5], Ting Huang[1], Yabin Zhang[1], Sin Man Lam[6,7], Huihui Li[5,*], Shiqian Qi[4,*], Jia Geng[2,3,*], and Kefeng Lu[1,*]

Affiliations:

[1]Department of Neurosurgery, State Key Laboratory of Biotherapy, West China Hospital, Sichuan University, Chengdu 610041, China

[2]Department of Laboratory Medicine, State Key Laboratory of Biotherapy, West China Hospital, Sichuan University, Chengdu 610041, China

[3]Department of Laboratory Medicine, State Key Laboratory of Biotherapy, Med-X Center for Manufacturing, West China Hospital, Sichuan University, Chengdu 610041, China

[4]Department of Urology, State Key Laboratory of Biotherapy, West China Hospital, Sichuan University, Chengdu 610041, China

[5]Department of Pathology, West China Second University Hospital, Sichuan University, Chengdu 610041, China

[6]State Key Laboratory of Molecular Developmental Biology, Institute of Genetics and Developmental Biology, Chinese Academy of Sciences, Beijing 100101, China.

[7]LipidALL Technologies Company Limited, Changzhou 213022, China.

[#]These authors contributed equally

*Correspondence to:

Kefeng Lu (lukf@scu.edu.cn), Jia Geng (geng.jia@scu.edu.cn),
Shiqian Qi. (qishiqian@scu.edu.cn) or Huihui Li. (lihuihui@scu.edu.cn)

Lead contact:

Kefeng Lu (lukf@scu.edu.cn)

Supplementary Figure S1

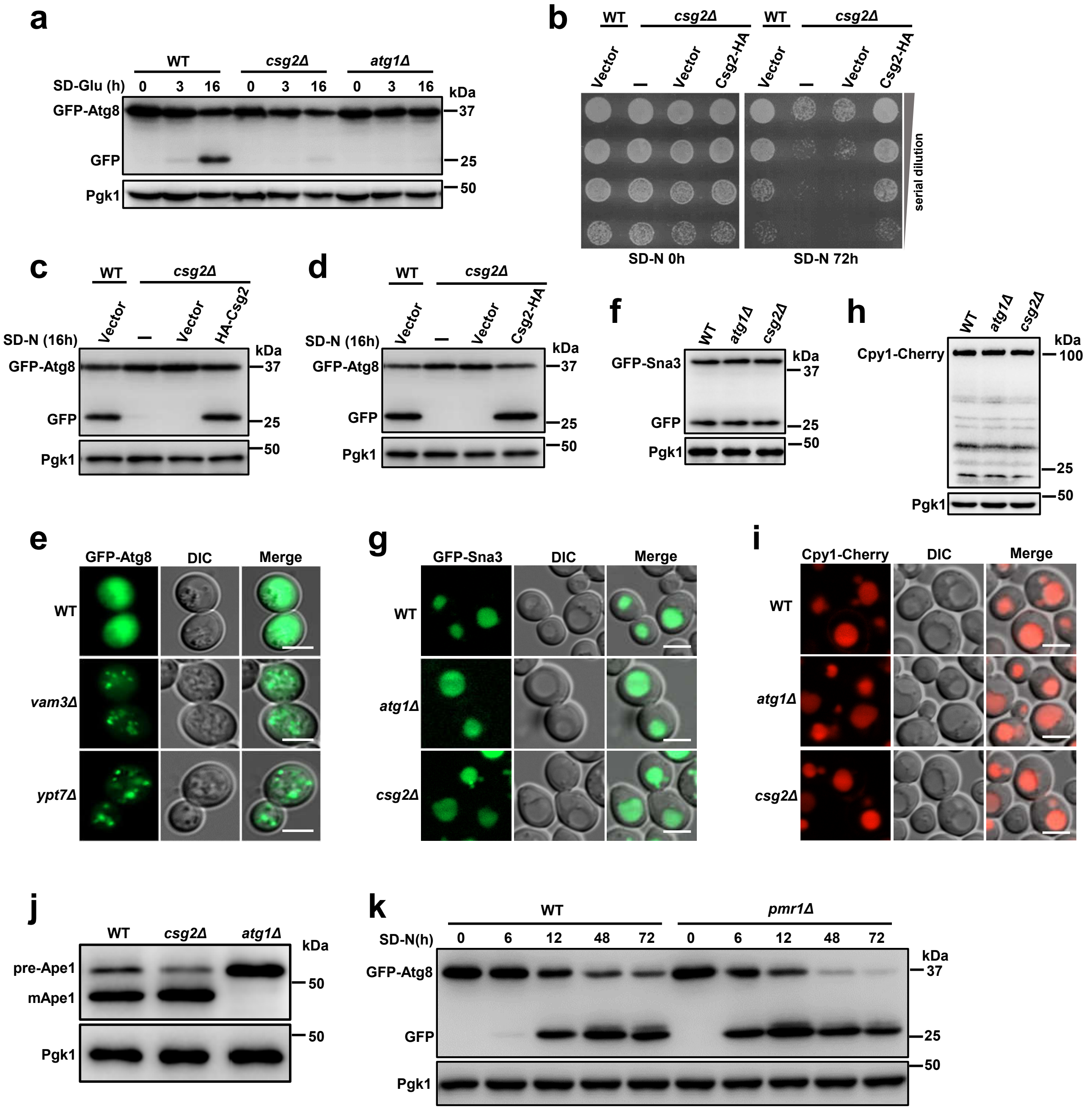

Supplementary Figure S1. Csg2 is essential for autophagy.

(a) Validation of degradation of autophagic substrate GFP-Atg8 in WT cells, csg2Δ cells and atg1Δ cells after starvation in synthetic minimal medium lacking glucose (SD-Glu) at the indicated times. The samples derive from the same experiment and gels were processed in parallel.

(b) WT cells and csg2Δ cells with or without exogenous expression of Csg2 were checked for cell viability before and after starvation in SD-N medium for 72 hours. The experiment was repeated independently for three times with similar results.

(c) and (d) WT cells and csg2Δ cells with or without expression of exogenous Csg2 (HA-Csg2 in c and Csg2-HA in d) were checked by GFP processing assays using autophagic substrate GFP-Atg8 following 16 hours of starvation in SD-N medium. The blots were probed with anti-GFP antibody and Pgk1 was used as a loading control. The samples derive from the same experiment and gels were processed in parallel.

(e) GFP-Atg8 was checked by fluorescence assays in indicated cells after starvation in SD-N medium for 3 hours in vam3Δ cells and ypt7Δ cells. The experiment was repeated independently for three times and representative images were shown. Scale bars: 5 μm.

(f) and (g) Degradation of endocytosis substrate GFP-Sna3 and its transportation in vacuoles in WT cells, csg2Δ cells and atg1Δ cells were investigated by western blot (f) and fluorescence assays (g). The blots were probed with anti-GFP antibody and Pgk1 was used as a loading control. The samples derive from the same experiment and gels were processed in parallel. The fluorescence assay was repeated independently for three times and representative images were shown. Scale bars: 5 μm.

(h) and (i) Vacuole trafficking of Cpy1-Cherry in WT cells, csg2Δ cells and atg1Δ cells was investigated by western blot (h) and fluorescence assays (i). The blots were probed with anti-Cherry antibody and Pgk1 was used as a loading control. The samples derive from the same experiment and gels were processed in parallel. The fluorescence assay was repeated independently for three times with and representative images were shown. Scale bars: 5 μm.

(j) Autophagic transfer of endogenous Ape1 in indicated yeast cells culture in rich medium was analyzed. The blots were probed with anti: Ape1 antibody and Pgk1 was used as a loading control. The samples derive from the same experiment and gels were processed in parallel.

(k) Degradation of autophagic substrate GFP-Atg8 was detected in WT cells and pmr1Δ cells after starvation in SD-N medium at the indicated times. The blots were probed with anti-GFP antibody and Pgk1 was used as a loading control. The samples derive from the same experiment and gels were processed in parallel.

Supplementary Figure S2

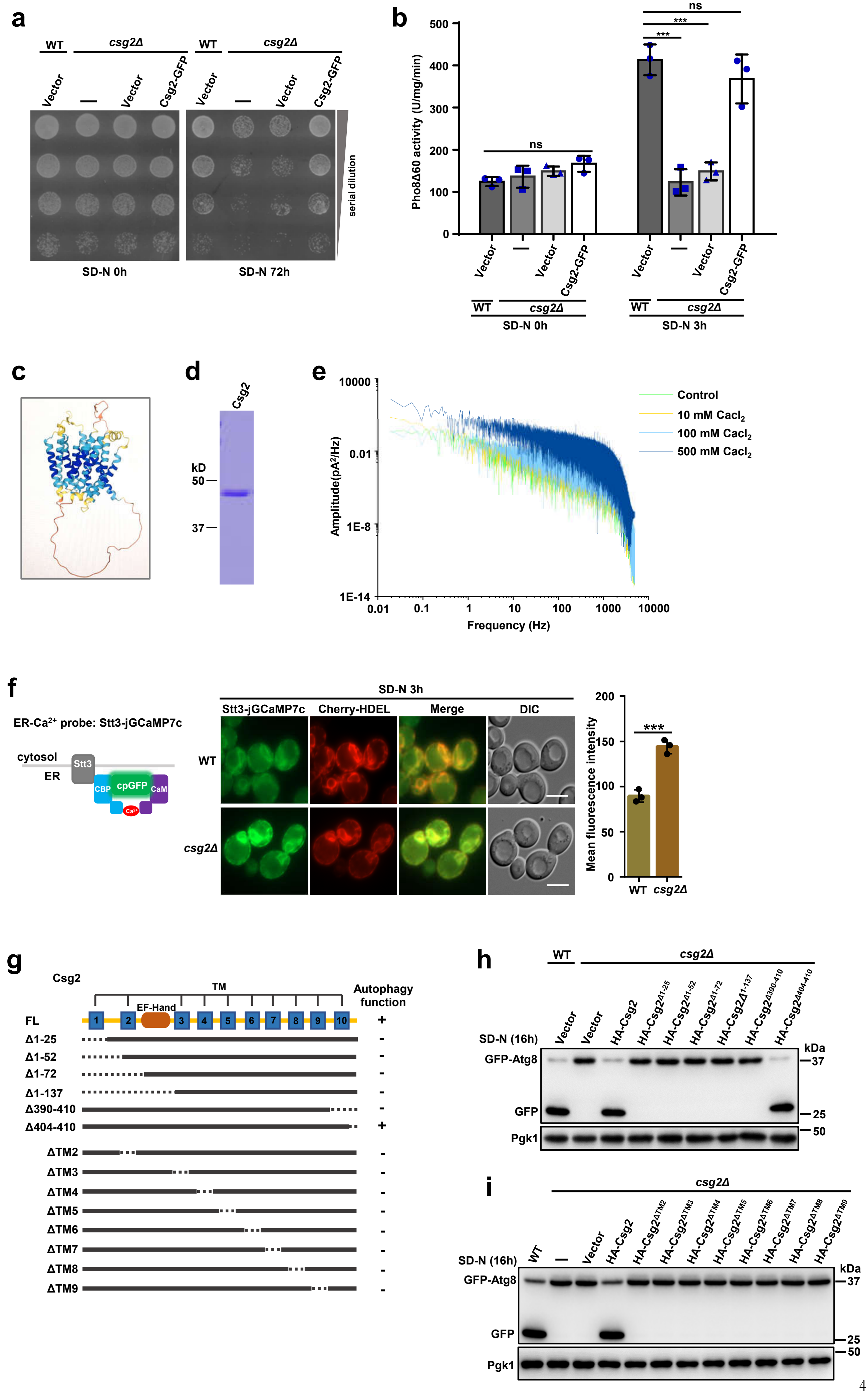

Supplementary Figure S2

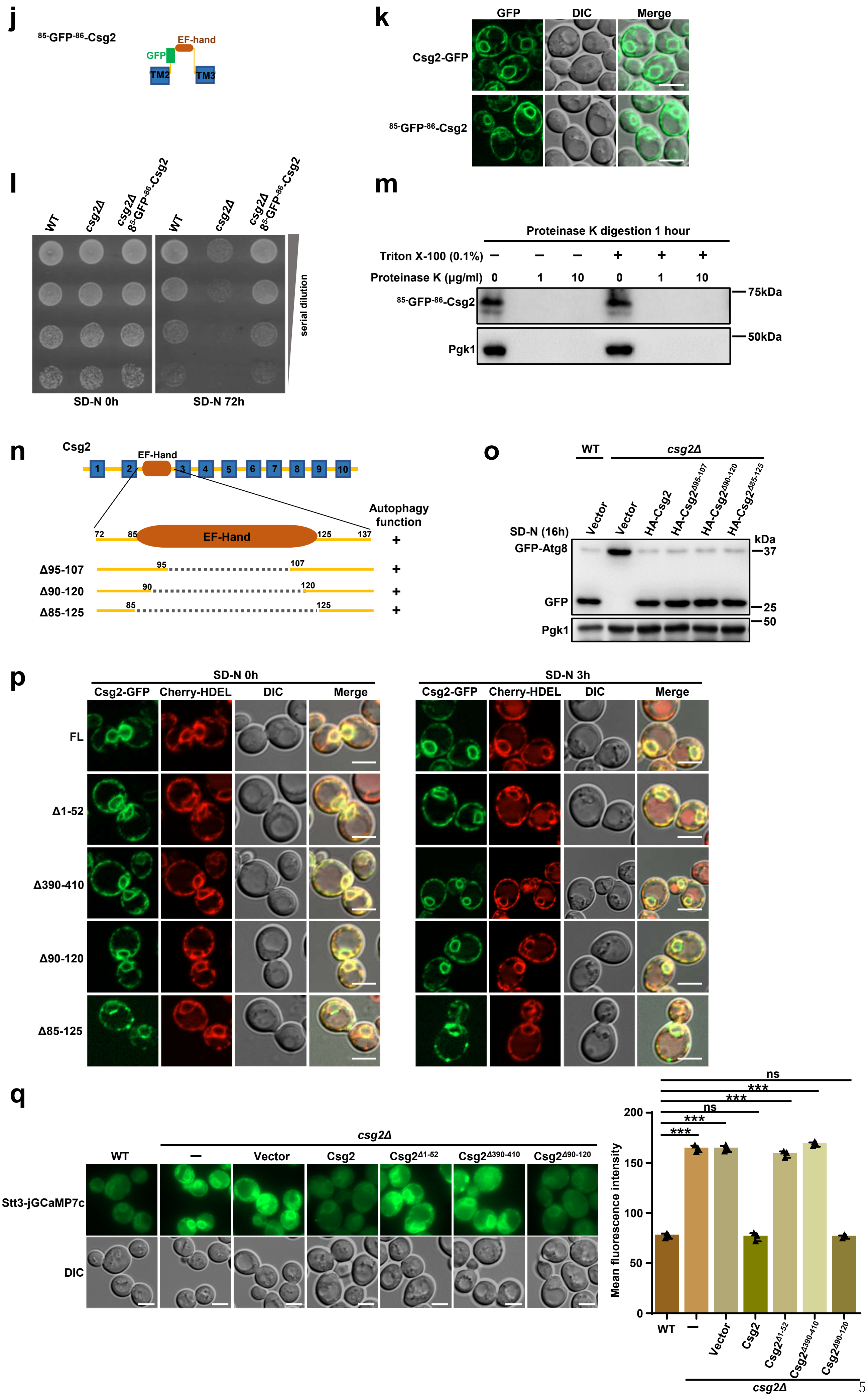

Supplementary Figure S2. The transmembrane domain of Csg2 is essential for autophagy.

(a) WT cells and csg2Δ cells with or without exogenous expression of Csg2-GFP were checked for cell viability before and after starvation in SD-N medium for 72 hours. The experiment was repeated independently for three times with similar results.

(b) Pho8Δ60 activities were measured before and after starvation in SD-N medium for 3 hours in indicated yeast cells by ALP assays. Two-tailed t-test, $n = 3$ independent experiments. SD-N 0h: p values: 0.5073, 0.0511,0.0554. SD-N 3h: p values: 0.000477, 0.000418, 0.3173. ***$p < 0.001$. ns: $p>0.05$, not significant. Error bars, mean ± SD.

(c) Schematic representation of Csg2 protein structure predicted by AlphaFold AI system.

(d) Coomassie blue staining of purified Csg2 protein (see Methods for detailed information of Csg2 expression and purification). The experiment was repeated independently at least three times with similar results.

(e) Power spectrum analysis of Csg2 channel activities with 10/100/500 mM Ca2+. The electrolyte buffer is (-cis) 30 mM NaCl -(-trans) 300 mM NaCl. The voltage was +20 mV. The experiment was repeated independently at least three times with similar results.

(f) ER calcium probe Stt3-jGCaMP7c and ER marker Cherry-HDEL were investigated by fluorescence assays in WT and csg2Δ cells after starvation in SD-N medium for 3 hours. Fluorescence parameters were set the same to compare the signal intensities between WT and csg2Δ cells. Representative images and fluorescence intensity quantification (right) were shown. Scale bars: 5 μm. Two-tailed t-test, $n = 3$ independent experiments. p=0.000811. ***$p < 0.001$. Error bars, mean ± SD.

(g) Schematic diagram of yeast Csg2 protein and indicated transmembrane (TM) truncates and their effects on the autophagic function of Csg2 was shown.

(h) and (i) Full-length or indicated TM truncated versions of Csg2 were expressed in csg2Δ cells and checked for their ability to restore the autophagic degradation of GFP-Atg8 after starvation in SD-N medium. The blots were probed with anti-GFP antibody and Pgk1 was used as a loading control. The samples derive from the same experiment and that gels were processed in parallel.

(j) Schematic representation of the Csg2 with N-terminal GFP-tagged EF-hand domain.

(k) Both C-terminal GFP-tagged Csg2 and Csg2 with N-terminal GFP-tagged EF-hand domain were localized at ER membranes. The experiment was repeated independently for three times and representative images were shown. Scale bars: 5 μm.

(l) Full-length or indicated TM truncated versions of Csg2 were expressed in csg2Δ cells and checked for their ability to restore the autophagic degradation of GFP-Atg8 after starvation in SD-N medium. The experiment was repeated independently for three times with similar results.

(m) Membrane topology experiments of Csg2. Cell lysates from WT cells expressing Csg2 with N-terminal GFP-tagged EF-hand domain were digested with proteinase K in the presence or absence of TritonX-100 followed by western blot analysis. Pgk1 served as a cytoplasmic protein control. The samples derive from the same experiment and gels were processed in parallel.

(n) Schematic diagram of yeast Csg2 protein and indicated EF-hand truncation and their effects on the autophagic function of Csg2 was shown.

(o) Full-length or indicated EF-hand truncated versions of Csg2 were expressed in csg2Δ cells and checked for their ability to restore the autophagic degradation of GFP-Atg8 after starvation in SD-N medium. The blots were probed with anti-GFP antibody and Pgk1 was used as a loading control. The samples derive from the same experiment and gels were processed in parallel.

(p) Co-location of the Full-length or indicated truncates of Csg2 with ER marker Cherry-HDEL was investigated by fluorescence assays before and after starvation in SD-N medium for 3 hours. The experiment was repeated independently for three times and representative images were shown. Scale bars: 5 μm.

(q) ER calcium probe Stt3-jGCaMP7c were investigated by fluorescence assays in indicated cells after starvation in SD-N medium for 3 hours. Fluorescence parameters were set the same to compare the signal intensities. Representative images and fluorescence intensity quantification (right) were shown. Scale bars: 5 μm. Two-tailed t-test, $n = 3$ independent experiments. p values: 3.5149E-06, 2.8328E-06, 0.7203, 3.8444E-06, 1.1196E-06, 0.6209. ***$p < 0.001$. ns: $p > 0.05$, not significant. Error bars, mean $\pm$ SD.

Supplementary Figure S3

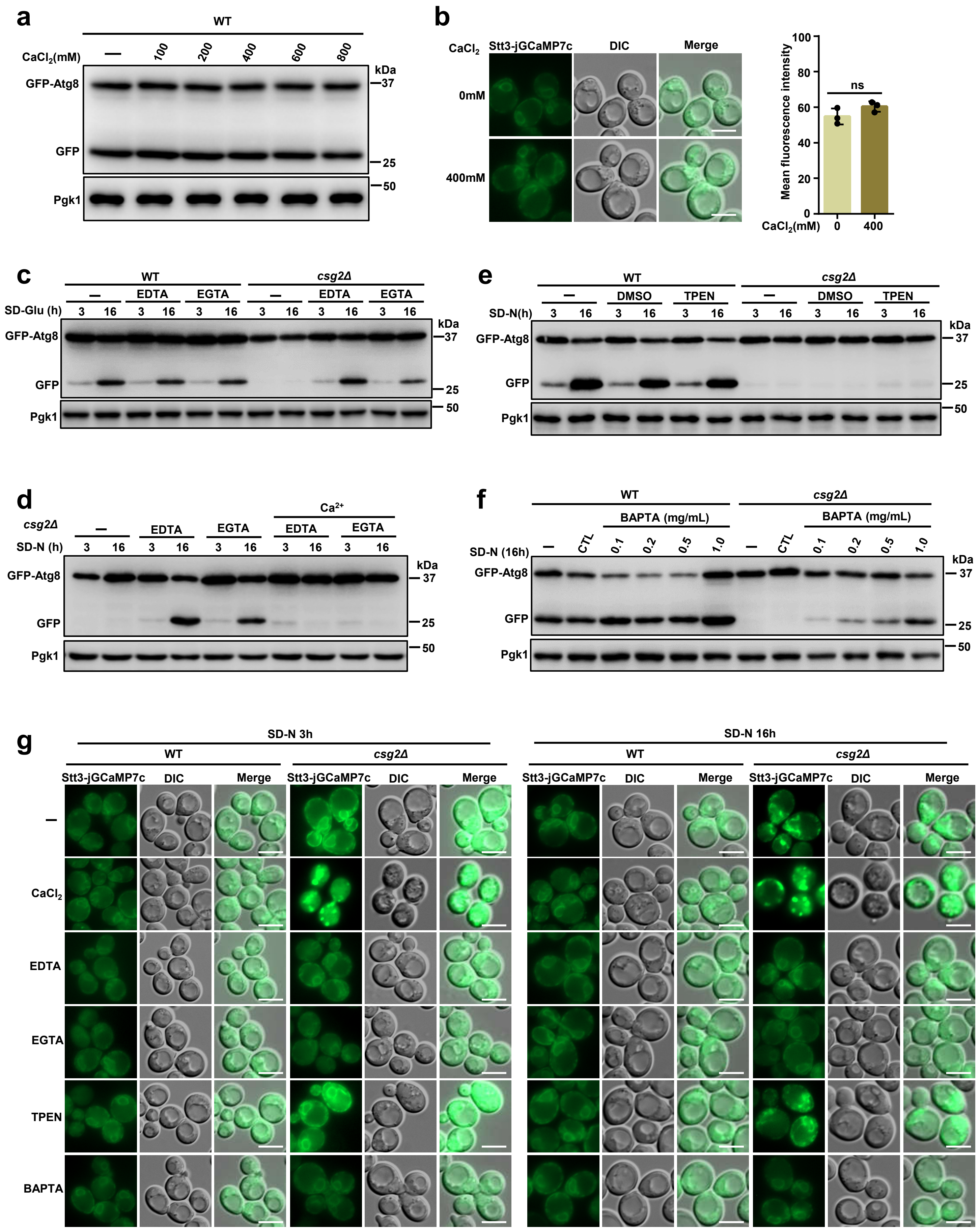

Supplementary Figure S3

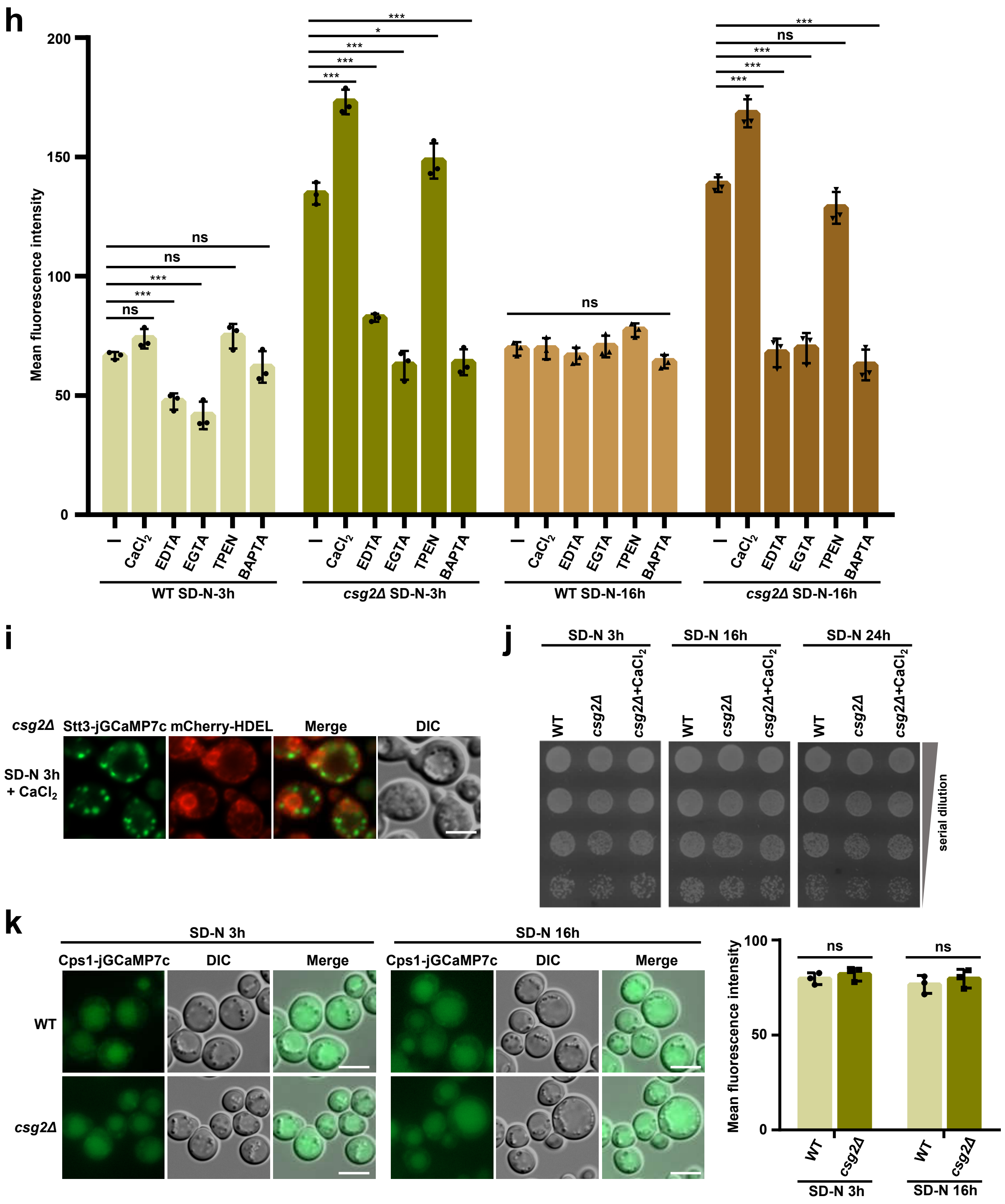

Supplementary Figure S3. Chelating of calcium restored autophagy in Csg2 deleted cells.

(a) GFP-Atg8 as autophagic substrate was detected for degradation after starvation in WT cells subject to SD-N medium with gradient concentrations of calcium. The blots were probed with anti-GFP antibody and Pgk1 was used as a loading control. The samples derive from the same experiment and gels were processed in parallel.

(b) ER calcium probe Stt3-jGCaMP7c were investigated by fluorescence assays in WT cells after starvation in SD-N medium with or without 400mM calcium for 3 hours. Fluorescence parameters were set the same to compare the signal intensities. Representative images and fluorescence intensity quantification (right) were shown. Scale bars: 5 μm. Two-tailed t-test, n = 3 independent experiments. p=0.1451. ns: p>0.05, not significant. Error bars, mean ± SD.

(c) Autophagy in WT yeast cells and csg2Δ cells was analyzed using GFP-Atg8 processing assays after starvation in SD-Glu medium with or without EDTA (5 mM) or EGTA (20 mM) at the indicated times. The blots were probed with anti-GFP antibody and Pgk1 was used as a loading control. The samples derive from the same experiment and gels were processed in parallel.

(d) Degradation of GFP-Atg8 in csg2Δ cells after starvation in SD-N medium with EDTA (5 mM), EGTA (20 mM), EDTA (5 mM) +Cacl2 (100 mM) or EGTA (20 mM) +Cacl2 (100 mM) was analyzed at the indicated times. The blots were probed with anti-GFP antibody and Pgk1 was used as a loading control. The samples derive from the same experiment and gels were processed in parallel.

(e) WT cells and csg2Δ cells treated with control DMSO or Zinc chelator TPEN (0.3 mM, solved in DMSO) were checked for autophagic degradation of GFP-Atg8 after starvation in SD-N medium at the indicated times. The blots were probed with anti-GFP antibody and Pgk1 was used as a loading control. The samples derive from the same experiment and gels were processed in parallel.

(f) WT cells and csg2Δ cells were checked for autophagic degradation of GFP-Atg8 after starvation in SD-N medium with control NaOH solute or Calcium chelator BAPTA (solved in NaOH). The blots were probed with anti-GFP antibody and Pgk1 was used as a loading control. The samples derive from the same experiment and gels were processed in parallel.

(g) and (h) ER calcium probe Stt3-jGCaMP7c were investigated by fluorescence assays in indicated cells after starvation in SD-N medium with different treatment for appointed times. Fluorescence parameters were set the same to compare the signal intensities. Representative images (g) and Fluorescence intensity quantification (h) were shown. Scale bars: 5 μm. Two-way repeated measures ANOVA corrected for multiple comparisons, n = 3 independent experiments. WT SD-N-3h: p values: 0.4895, 0.0003, 0.0001, 0.3263, 0.8248; csg2Δ SD-N-3h: p values: 5.70E-05, 5.1940E-05, 8.1202E-05, 0.0157, 6.8717E-05; WT SD-N-16h: p values: 0.9954, 0.9737, 0.9999, 0.3778, 0.7555; csg2Δ SD-N-16h: p values: 0.0001, 5.5930E-05, 7.2823E-05, 0.1670, 5.2002E-05. *p < 0.05, **p < 0.01, ***p < 0.001. ns: p>0.05, not significant. Error bars, mean ± SD.

(i) ER calcium probe Stt3-jGCaMP7c and ER marker Cherry-HDEL in csg2Δ cells were detected colocalization after starvation in SD-N medium with 100 mM Cacl2 for 3 hours. The experiment was repeated independently for three times and representative images were shown. Scale bars: 5 μm.

(j) csg2Δ cells were checked for cell viability after starvation in SD-N medium with 100 mM Cacl2 in indicated time points. The experiment was repeated independently for three times with similar results.

(k) Vacuolar calcium probe Cps1-jGCaMP7c was investigated after starvation in SD-N medium for different times by fluorescence assays. Fluorescence parameters were set the same to compare the signal intensities. Representative images and Fluorescence intensity quantification (right) were shown. Scale bars: 5 μm. Two-tailed t-test, n = 3 independent experiments. p values:0.4321, 0.4928. ns: p>0.05, not significant. Error bars, mean ± SD.

Supplementary Figure S4

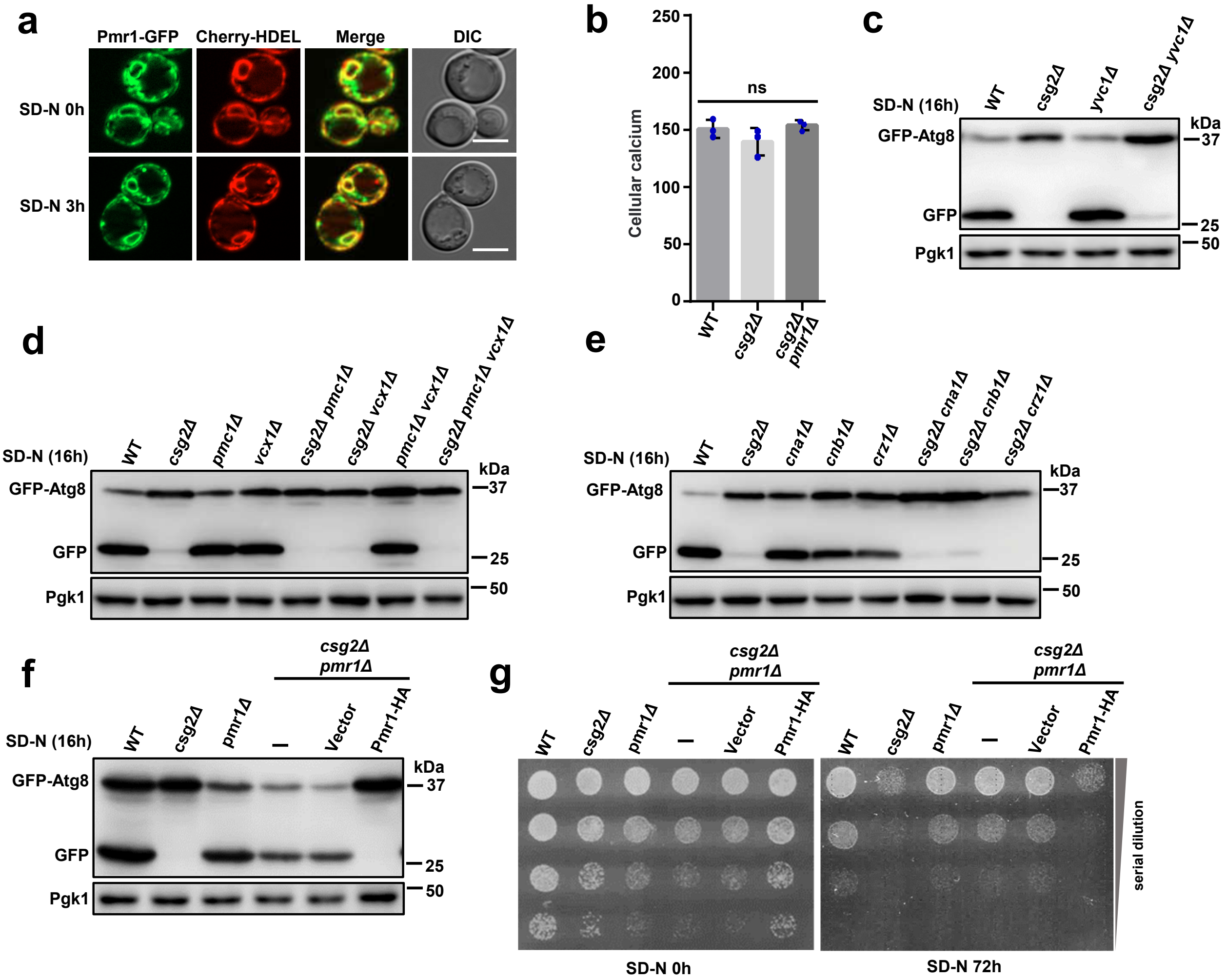

Supplementary Figure S4. Calcium accumulation in ER was responsible for blockage of autophagy in csg2Δ yeast cells.

(a) Colocalization of Pmr1-GFP and ER marker Cherry-HDEL in WT yeast cells was analyzed by fluorescence assays before and after starvation in SD-N medium. The experiment was repeated independently for three times and representative images were shown. Scale bars: 5 μm.

(b) Calcium was checked in designated cells after starvation SD-N medium for 3 hours by quantitative colorimetry via Ca2+ reacting with o-phenolphthalein complex ketone to produce a violet-blue complex (see Methods for details). Two-tailed t-test, n = 3 independent experiments. p values: 0.2501, 0.5563. ns: $p>0.05$, not significant. Error bars, mean ± SD.

(c) and (d) WT cells and csg2Δ cells with or without deletion of vacuole calcium channel were analyzed for the autophagic degradation of GFP-Atg8 after starvation in SD-N medium. The blots were probed with anti-GFP antibody and Pgk1 was used as a loading control. The samples derive from the same experiment and that gels were processed in parallel.

(e) WT cells and csg2Δ cells with or without deletion of cytoplasmic calcium signaling factors were analyzed for the autophagic degradation of GFP-Atg8 after starvation in SD-N medium. The blots were probed with anti-GFP antibody and Pgk1 was used as a loading control. The samples derive from the same experiment and gels were processed in parallel.

(f) Autophagic degradation of GFP-Atg8 was blocked in csg2Δ pmr1Δ cells with exogenous expression of Pmr1 after starvation in SD-N medium. The blots were probed with anti-GFP antibody and Pgk1 was used as a loading control. The samples derive from the same experiment and gels were processed in parallel.

(g) Expression of Pmr1 blocked starvation resistance in csg2Δ pmr1Δ yeast cells. Indicated yeast cells were checked for cell viability before and after starvation in SD-N medium. The experiment was repeated independently for three times with similar results.

Supplementary Figure S5

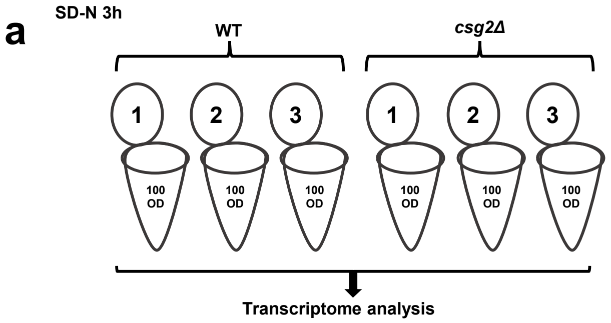

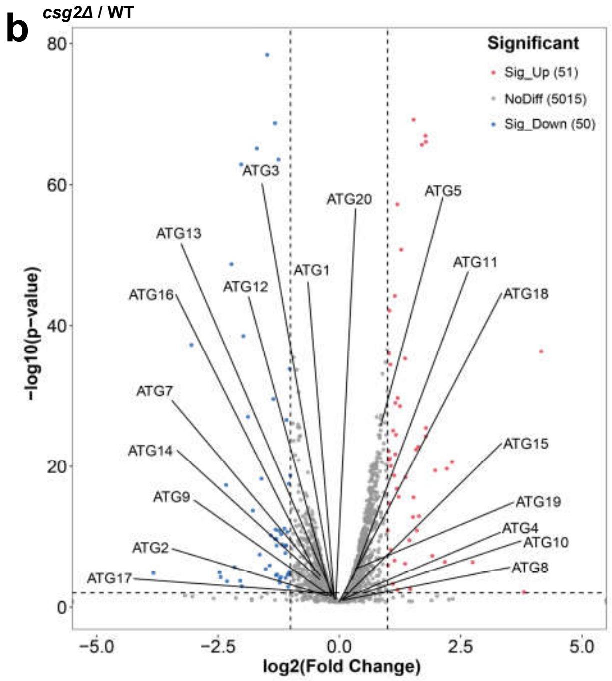

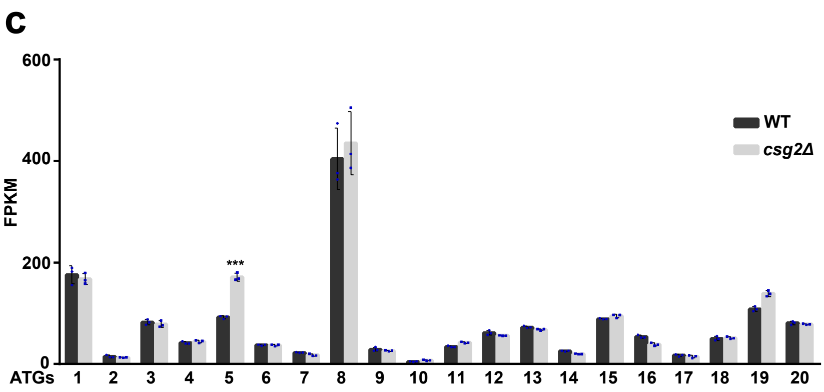

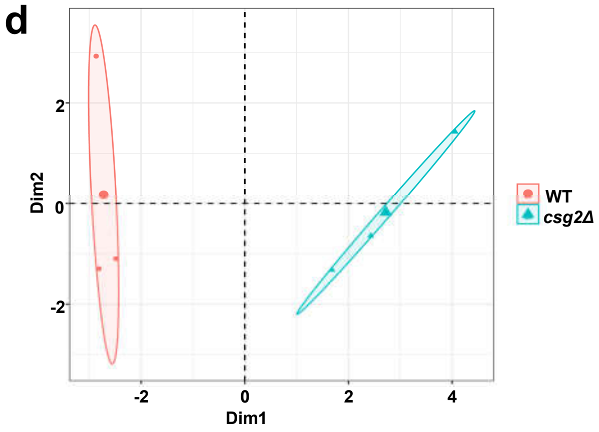

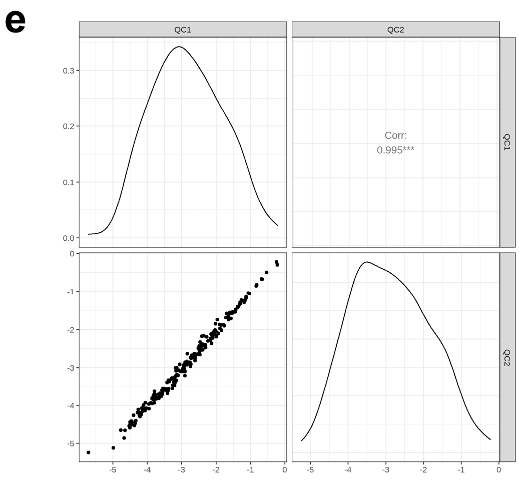

f

Lipids	abbreviation
diacylglycerol	DAG
triacylglycerol	TAG
sterol	sterol
steryl ester	steryl ester
PhytoCeramide	PhytoCer
Phytosphingosine	PHS
phosphatidylcholine	PC
lyso-phosphatidylcholine	LPC
phosphatidylethanolamine	PE
lyso-phosphatidylethanolamine	LPE
phosphatidic acid	PA
lyso-phosphatidic acid	LPA
phosphatidylinositol	PI
lyso-phosphatidylinositol	LPI
phosphatidylserine	PS
lyso-phosphatidylserine	LPS
phosphatidylglycerol	PG
free fatty acid	FFA

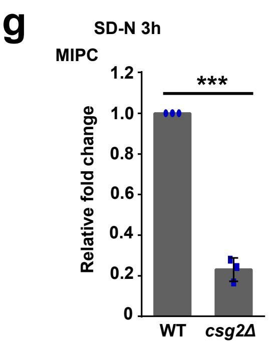

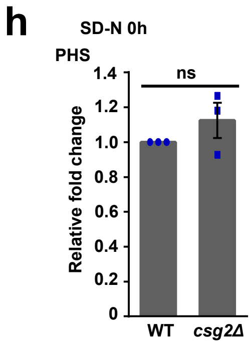

Supplementary Figure S5. The mRNA transcription of ATG genes maintained normal in csg2Δ cells.

(a) A schematic diagram of the transcriptome analysis of WT and csg2Δ yeast cells after starvation in SD-N medium. Experiments were set up in three biologically independent samples.

(b) and (c) Deletion of Csg2 had no effect on transcription of autophagy-related genes shown by volcano plot and by statistics quantification analysis. Two-tailed t-test, $n = 3$ biologically independent samples. Error bars, mean $\pm$ SD. For details see Supplementary Data.

(d) PCA analysis showed that the two groups of samples had no significant outliers and were distinguished in principal components. Ellipse showing 95% distribution interval.

(e) A high correlation coefficient > 0.99 between the two QC samples demonstrated good consistency of signal during the mass spectrometric runs and a good quality in the mass spectrometric data.

(f) Lipid abbreviations.

(g) MIPC decreased in csg2Δ cells after starvation in SD-N medium for 3 hours. Two-tailed t-test, $n = 3$ biologically independent samples. p=2.0046E-05. ***$p < 0.001$. Error bars, mean $\pm$ SD.

(h) PHS was not increased in csg2Δ cells cultured in nutrient rich medium. Two-tailed t-test, $n = 3$ biologically independent samples. p=0.2838. ns: $p>0.05$, not significant. Error bars, mean $\pm$ SD.

Supplementary Figure S6

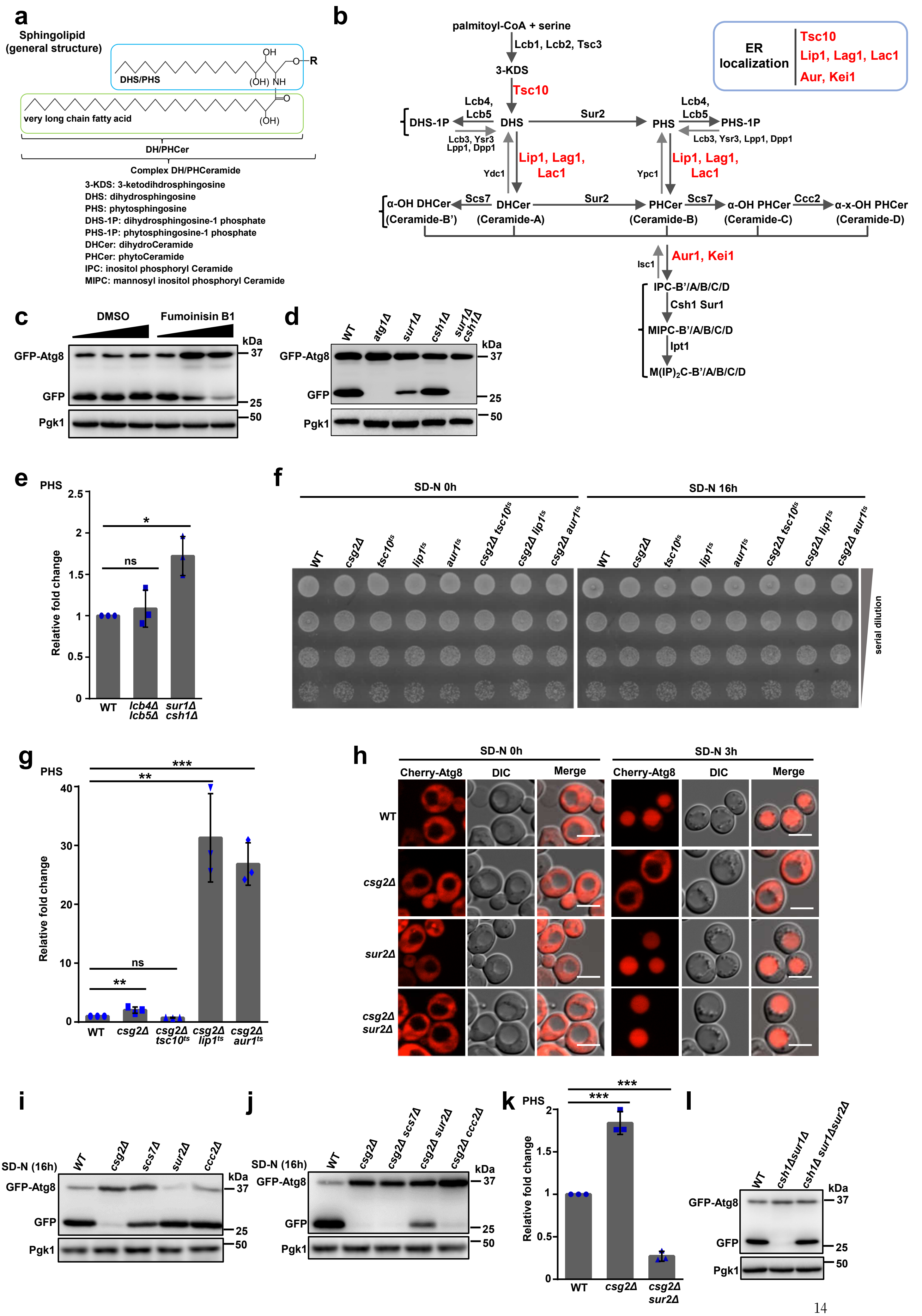

Supplementary Figure S6

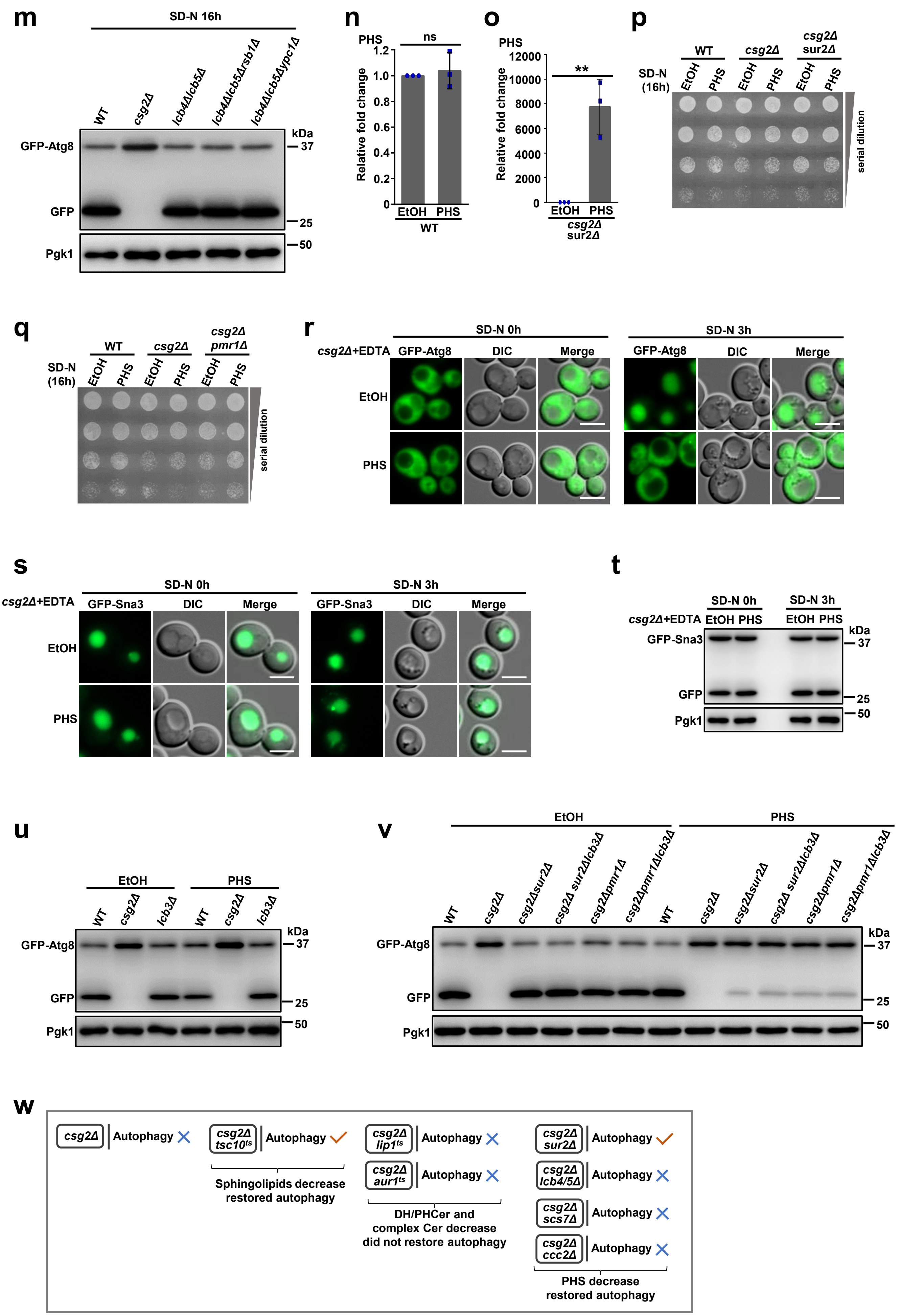

Supplementary Figure S6. PHS was responsible for blockage of autophagy in csg2Δ cells.

(a) Schematic diagram of the structures of sphingolipids.

(b) Schematic diagram of the sphingolipid synthesis pathway in yeast cells.

(c) Degradation of GFP-Atg8 was detected in WT cells after starvation in SD-N medium with gradient concentration the ceramide synthesis inhibitor Fumoinisin B1 (0.01mg/mL, 0.05mg/mL, 0.1mg/mL) dissolved in DMSO at 16 hours. The blots were probed with anti-GFP antibody and Pgk1 was used as a loading control. The samples derive from the same experiment and that gels were processed in parallel.

(d) Autophagy was blocked in yeast cells with double deletion but not single deletion of Csh1 and Sur1. The blots were probed with anti-GFP antibody and Pgk1 was used as a loading control. The samples derive from the same experiment and gels were processed in parallel.

(e) 100OD yeast cells starved in SD-N medium for 3 hours were collected for phytosphingosine lipid analysis. Two-tailed t-test, n = 3 biologically independent samples. p values: 0.4488, 0.0142. *p < 0.05. ns: p>0.05, not significant. Error bars, mean ± SD.

(f) The cell viabilities of indicated cells were detected before or after starvation in SD-N medium for 16 hours at 37° C. The experiment was repeated independently for three times with similar results.

(g) Phytosphingosine levels were analyzed in tsc10ts, lip1ts and aur1ts yeast cells combining with Csg2 deletion after starvation in SD-N medium for 3 hours at nonpermissive 37° C. Two-tailed t-test, n = 3 biologically independent samples. p values: 0.0092, 0.2705, 0.0021, 0.00024. **p < 0.01, ***p < 0.001. ns: p>0.05, not significant. Error bars, mean ± SD.

(h) Cellular distribution of autophagy marker Cherry-Atg8 in WT cells, csg2Δ cells, sur2Δ cells and csg2Δsur2Δ cells was analyzed by fluorescence assays before and after starvation in SD-N medium. The experiment was repeated independently for three times and representative images were shown. Scale bars: 5 μm.

(i) and (j) Autophagic degradation of GFP-Atg8 in indicated yeast cells was analyzed after starvation in SD-N medium for 16 hours. The blots were probed with anti-GFP antibody and Pgk1 was used as a loading control. The samples derive from the same experiment and gels were processed in parallel.

(k) 100OD WT cells, csg2Δ and csg2 Δsur2Δ yeast cells were collected after 3 hours of starvation in SD-N medium and subject to phytosphingosine lipid analysis. Two-tailed t-test, n = 3 biologically independent samples. p values: 0.000424, 2.5787E-05. ***p < 0.001. Error bars, mean ± SD.

(l) Blocked autophagy in csh1Δ sur1Δ cells was regained by further deletion of Sur2. The blots were probed with anti-GFP antibody and Pgk1 was used as a loading control. The samples derive from the same experiment and that gels were processed in parallel.

(m) GFP-Atg8 was checked for degradation after nitrogen starvation in different mutant cells after starvation. The blots were probed with anti-GFP antibody and Pgk1 was used as a loading control. The samples derive from the same experiment and gels were processed in parallel.

(n) PHS were analyzed in WT cells after starvation in SD-N medium added with PHS (5 μM, EtOH as control) for 3 hours. Two-tailed t-test, n = 3 biologically independent samples. p=0.6640. ns: p>0.05, not significant. Error bars, mean ± SD.

(o) PHS accumulated significantly after nitrogen starvation added exogenous PHS (5 μM, EtOH as control) for 3 hours in csg2Δ sur2Δ yeast cells. Two-tailed t-test, n = 3 biologically independent samples. p=0.004073. **p < 0.01. Error bars, mean ± SD.

(p) and (q) WT cells, csg2Δ cells, csg2Δ sur2Δ cells and csg2Δ pmr1Δ cells after starvation in SD-N medium with PHS (5 μM, EtOH as control) for 16 hours were analyzed by cell viability assays. The experiment was repeated independently for three times with similar results.

(r) GFP-Atg8 under control of endogenous ATG8 gene promoter as autophagic marker was investigated by fluorescence examination in csg2Δ cells before and after starvation in SD-N medium with EDTA (5 mM) and PHS (5 μM, EtOH as control). The experiment was repeated independently for three times and representative images were shown. Scale bars: 5 μm.

(s) and (t) Endocytosis in csg2Δ cells added EDTA was unaffected before and after starvation in SD-N medium with addition of PHS (5 μM, EtOH as control) shown by fluorescence assays (s) and western blot (t). The experiment was repeated independently for three times and representative images were shown. Scale bars: 5 μm. The blots were probed with anti-GFP antibody and Pgk1 was used as a loading control. The samples derive from the same experiment and gels were processed in parallel.

(u) and (v) GFP-Atg8 was checked for degradation after starvation in SD-N medium with PHS (5 μM, EtOH as control) for 16 hours in the indicated yeast cells. The blots were probed with anti-GFP antibody and Pgk1 was used as a loading control. The samples derive from the same experiment and gels were processed in parallel.

(w) In csg2Δ cells, autophagy was blocked; in csg2Δ tsc10ts cells (low sphingolipids), autophagy was restored, indicating that accumulation but not decrease of certain sphingolipid(s) is the reason for autophagy blockage caused by Csg2 deletion. In csg2Δ lip1ts or csg2Δ aur1ts cells (high DHS/PHS, low complex Cers), autophagy was not restored, indicating that it is the accumulation of PHS or DHS (or phos-DHS/PHS) but not the downstream sphingolipid(s) that causes autophagy blockage. Further deletion of Sur2 but not Lcb4/5, Ssc7 or Ccc2 can restore autophagy in csg2Δ cells, indicating that it is the accumulation of PHS that causes autophagy blockage in csg2Δ cells.

Supplementary Figure S7

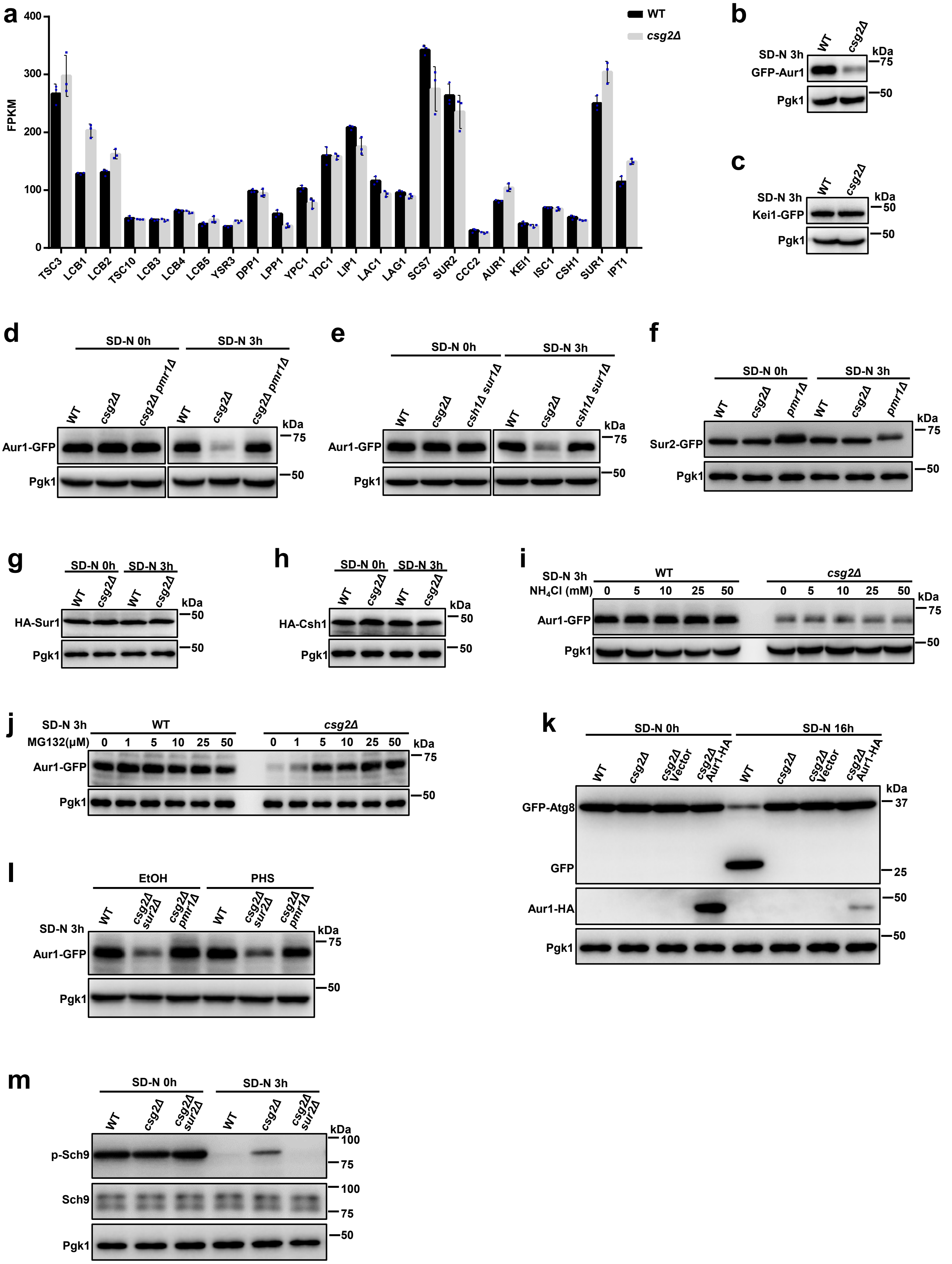

Supplementary Figure S7. Aur1 was disrupted in csg2Δ yeast cells.

(a) mRNA levels of enzymes involved in yeast sphingolipid synthesis pathway were not changed in csg2Δ cells. Two-tailed t-test, n = 3 biologically independent samples. Error bars, mean ± SD. For details see Supplementary Data.

(b) N-terminal GFP-tagged Aur1 was expressed and analyzed in WT cells and csg2Δ cells after starvation in SD-N medium. The blots were probed with anti-GFP antibody and Pgk1 was used as a loading control. The samples derive from the same experiment and gels were processed in parallel.

(c) WT cells and csg2Δ cells expressing Kei1-GFP were subject to starvation in SD-N medium followed by protein analysis. The blots were probed with anti-GFP antibody and Pgk1 was used as a loading control. The samples derive from the same experiment and gels were processed in parallel.

(d) Aur1 protein level was restored by deletion of Pmr1 in csg2Δ cells after starvation. The blots were probed with anti-GFP antibody and Pgk1 was used as a loading control. The samples derive from the same experiment and gels were processed in parallel.

(e) Expressed Aur1-GFP in WT cells and csh1Δ sur1Δ cells were analyzed before and after starvation in SD-N medium by immunoblotting. The blots were probed with anti-GFP antibody and Pgk1 was used as a loading control. The samples derive from the same experiment and gels were processed in parallel.

(f) Expression of Sur2-GFP in WT cells, csg2Δ cells and pmr1Δ cells were checked before and after starvation by protein analysis. The blots were probed with anti-GFP antibody and Pgk1 was used as a loading control. The samples derive from the same experiment and gels were processed in parallel.

(g) and (h) N-terminal HA-tagged Sur1 and Csh1 was expressed in WT cells and csg2Δ cells and inspected by immunoblotting. The blots were probed with anti-HA antibody and Pgk1 was used as a loading control. The samples derive from the same experiment and gels were processed in parallel.

(i) Aur1-GFP in WT cells and csg2Δ cells was analyzed after treatment in SD-N medium added gradient autophagic inhibitor (NH4Cl). The blots were probed with anti-GFP antibody and Pgk1 was used as a loading control. The samples derive from the same experiment and gels were processed in parallel.

(j) The addition of a gradient concentrations of proteasome inhibitor (MG132) in csg2Δ cells in SD-N medium resulted in Aur1 protein accumulation. The blots were probed with anti-GFP antibody and Pgk1 was used as a loading control. The samples derive from the same experiment and gels were processed in parallel.

(k) Autophagy was not rescued by overexpression of Aur1 in csg2Δ cells. The blots were probed with anti-GFP antibody and HA antibody, Pgk1 was used as a loading control. The samples derive from the same experiment and gels were processed in parallel.

(l) Deletion of Pmr1 rather than Sur2 could regain the protein levels of Aur1 in csg2Δ cells starved in SD-N medium with PHS (5 μM, EtOH as control) for 3 hours. The blots were probed with anti-GFP antibody and Pgk1 was used as a loading control. The samples derive from the same experiment and gels were processed in parallel.

(m) Accumulation of PHS in csg2Δ cells caused activation of TORC1, which was reversed by further Sur2 deletion. The phosphorylation levels of Sch9 and the total protein levels were analyzed. Pgk1 was used as a loading control. The samples derive from the same experiment and gels were processed in parallel.

Supplementary Table S1
Yeast strains used in this study

MATa ura3Δ0 leu2Δ0 his3Δ1 met15Δ0	EUROSCARF	BY4741(WT)
BY4741 *csg2::hphNT1*	This paper	*csg2Δ*
BY4741 *atg1::natNT2*	This paper	*atg1Δ*
BY4741 *pmr1::natNT2*	This paper	*pmr1Δ*
BY4741 *spf1::kanMX6*	This paper	*spf1Δ*
BY4741 *pmc1::HIS3MX6*	This paper	*pmc1Δ*
BY4741 *vcx1::HIS3MX6*	This paper	*vcx1Δ*
BY4741 *yvc1::natNT2*	This paper	*yvc1Δ*
BY4741 *mid1::kanMX6*	This paper	*mid1Δ*
BY4741 *rch1::kanMX6*	This paper	*rch1Δ*
BY4741 *ecm7::kanMX6*	This paper	*ecm7Δ*
BY4741 *cch1::kanMX6*	This paper	*cch1Δ*
BY4741 *atg8::kanMX6*	This paper	*atg8Δ*
BY4741 *vam3::kanMX6*	This paper	*vam3Δ*
BY4741 *ypt7::kanMX6*	This paper	*ypt7Δ*
BY4741 *csg2::hphNT1 pmc1::HIS3MX6*	This paper	*csg2Δpmc1Δ*
BY4741 *csg2::hphNT1 vcx1::HIS3MX6*	This paper	*csg2Δvcx1Δ*
BY4741 *pmc1::HIS3MX6 vcx1::natNT2*	This paper	*pmc1Δvcx1Δ*
BY4741 *csg2::hphNT1 pmc1::HIS3MX6 vcx1::natNT2*	This paper	*csg2Δpmc1Δvcx1Δ*
BY4741 *csg2::hphNT1 pmr1::natNT2*	This paper	*csg2Δpmr1Δ*
BY4741 *cna1::natNT2*	This paper	*cna1Δ*
BY4741 *cnb1::natNT2*	This paper	*cnb1Δ*
BY4741 *csg2::hphNT1 cna1::natNT2*	This paper	*csg2Δcna1Δ*
BY4741 *csg2::hphNT1 cnb1::natNT2*	This paper	*csg2Δcnb1Δ*
BY4741 *crz1::natNT2*	This paper	*crz1Δ*
BY4741 *csg2::hphNT1 crz1::natNT2*	This paper	*csg2Δcrz1Δ*
BY4741 *csg2::hphNT1 yvc1::natNT2*	This paper	*csg2Δyvc1Δ*
BY4741 *lcb4::natNT2*	This paper	*lcb4Δ*
BY4741 *lcb5::natNT2*	This paper	*lcb5Δ*
BY4741 *lcb4::natNT2*	This paper	*lcb4Δlcb5Δ*

lcb5::HIS3MX6		
BY4741 *csg2::hphNT1 lcb4::HIS3MX6*	This paper	*csg2Δlcb4Δ*
BY4741 *csg2::hphNT1 lcb5::HIS3MX6*	This paper	*csg2Δlcb5Δ*
BY4741 *csg2::hphNT1 lcb4::HIS3MX6 lcb5::natNT2*	This paper	*csg2Δlcb4Δlcb5Δ*
BY4741 *sur2::natNT2*	This paper	*sur2Δ*
BY4741 *csg2::hphNT1 sur2::natNT2*	This paper	*csg2Δsur2Δ*
BY4741 *scs7::natNT2*	This paper	*scs7Δ*
BY4741 *ccc2::natNT2*	This paper	*ccc2Δ*
BY4741 *csg2::hphNT1 scs7::natNT2*	This paper	*csg2Δscs7Δ*
BY4741 *csg2::hphNT1 ccc2::natNT2*	This paper	*csg2Δccc2Δ*
BY4741 $tsc10^{ts}$*::URA3*	Dr. Peter C. Stirling lab	$tsc10^{ts}$
BY4741 $lip1^{ts}$*::URA3*	Dr. Peter C. Stirling lab	$lip1^{ts}$
BY4741 $aur1^{ts}$*::URA3*	Dr. Peter C. Stirling lab	$aur1^{ts}$
BY4741 $tsc10^{ts}$*::URA3 csg2::hphNT1*	This paper	$tsc10^{ts}$*csg2Δ*
BY4741 $lip1^{ts}$*::URA3 csg2::hphNT1*	This paper	$lip1^{ts}$*csg2Δ*
BY4741 $aur1^{ts}$*::URA3 csg2::hphNT1*	This paper	$aur1^{ts}$*csg2Δ*
BY4741 *csh1::hphNT1*	This paper	*csh1Δ*
BY4741 *sur1::hphNT1*	This paper	*sur1Δ*
BY4741 *csh1::hphNT1 sur1::natNT2*	This paper	*csh1Δsur1Δ*
BY4741 *lcb4::natNT2 lcb5::HIS3MX6 rsb1::hphNT1*	This paper	*lcb4Δlcb5Δrsb1Δ*
BY4741 *lcb4::natNT2 lcb5::HIS3MX6 ypc1::hphNT1*	This paper	*lcb4Δlcb5Δypc1Δ*
BY4741 *lcb3::HIS3MX6*	This paper	*lcb3Δ*
BY4741 *csg2::hphNT1 sur2::natNT2 lcb3::HIS3MX6*	This paper	*csg2Δsur2Δlcb3Δ*
BY4741 *csg2::hphNT1 pmr1::natNT2 lcb3::HIS3MX6*	This paper	*csg2Δpmr1Δlcb3Δ*

Italic formatting means gene replacement, deletion or mutation.

Supplementary Table S2
Expression constructs used in this study

p413-prADH1-GFP-Atg8	This paper	GFP-Atg8
pUG36--prMET25-GFP-50Q	This paper	GFP-50Q
p413-prADH1-RFP-Ape1	This paper	RFP-Ape1
p413-prADH1-Pho8Δ60	This paper	Pho8Δ60
pUG34-prMET25-GFP-Sna3	This paper	GFP-Sna3
p416-prADH1-Cpy1-Cherry	This paper	Cpy1-Cherry
pTPI1-Cherry-HDEL	Dr. Zhiping Xie lab	Cherry-HDEL
p415-prADH1-HA-Csg2	This paper	HA-Csg2
p415-prADH1-Csg2-HA	This paper	Csg2-HA
p415-prADH1-HA-Csg2-Δ1-25	This paper	HA-Csg2$^{\Delta 1\text{-}25}$
p415-prADH1-HA-Csg2-Δ1-52	This paper	HA-Csg2$^{\Delta 1\text{-}52}$
p415-prADH1-HA-Csg2-Δ1-72	This paper	HA-Csg2$^{\Delta 1\text{-}72}$
p415-prADH1-HA-Csg2-Δ1-137	This paper	HA-Csg2$^{\Delta 1\text{-}137}$
p415-prADH1-HA-Csg2-Δ390-410	This paper	HA-Csg2$^{\Delta 390\text{-}410}$
p415-prADH1-HA-Csg2-Δ404-410	This paper	HA-Csg2$^{\Delta 404\text{-}410}$
p415-prADH1-HA-Csg2-Δ95-107	This paper	HA-Csg2$^{\Delta 95\text{-}107}$
p415-prADH1-HA-Csg2-Δ90-120	This paper	HA-Csg2$^{\Delta 90\text{-}120}$
p415-prADH1-HA-Csg2-Δ85-125	This paper	HA-Csg2$^{\Delta 85\text{-}125}$
p415-prADH1-Csg2-GFP	This paper	Csg2-GFP
p415-prADH1-Csg2-Δ1-52-GFP	This paper	Csg2-Δ1-52-GFP
p415-prADH1-Csg2-Δ390-410-GFP	This paper	Csg2-Δ390-410-GFP
p415-prADH1-Csg2-Δ90-120-GFP	This paper	Csg2-Δ90-120-GFP
p415-prADH1-Csg2-Δ85-125-GFP	This paper	Csg2-Δ85-125-GFP
p416-prADH1-Pmr1-HA	This paper	Pmr1-HA
p416-prADH1-Pmr1^{D53A}-HA	This paper	Pmr1^{D53A}-HA
p415-prADH1-Cherry-Atg8	This paper	Cherry-Atg8
p416-prADH1-Pmr1-GFP	This paper	Pmr1-GFP
p415-prADH1-Sur2-HA	This paper	Sur2-HA
p415-prADH1-Tsc10-GFP	This paper	Tsc10-GFP
p415-prADH1-Lip1-GFP	This paper	Lip1-GFP
p415-prADH1-Aur1-GFP	This paper	Aur1-GFP
p416-prADH1-Kei1-HA	This paper	Kei1-HA
p415-prADH1-GFP-Aur1	This paper	GFP-Aur1
p416-prADH1-Kei1-GFP	This paper	Kei1-GFP
p415-prADH1-Stt3-jGCamP7c	This paper	Stt3-jGCaMP7c
p416-prADH1-Stt3-jGCamP7c	This paper	Stt3-jGCaMP7c
pFastBac-Dual-Csg2-TEV-2*Strep-Flag	This paper	Csg2-TEV-Strep

p415-prADH1-HA-Csg2-ΔTM2	This paper	HA-Csg2$^{\Delta TM2}$
p415-prADH1-HA-Csg2-ΔTM3	This paper	HA-Csg2$^{\Delta TM3}$
p415-prADH1-HA-Csg2-ΔTM4	This paper	HA-Csg2$^{\Delta TM4}$
p415-prADH1-HA-Csg2-ΔTM5	This paper	HA-Csg2$^{\Delta TM5}$
p415-prADH1-HA-Csg2-ΔTM6	This paper	HA-Csg2$^{\Delta TM6}$
p415-prADH1-HA-Csg2-ΔTM7	This paper	HA-Csg2$^{\Delta TM7}$
p415-prADH1-HA-Csg2-ΔTM8	This paper	HA-Csg2$^{\Delta TM8}$
p415-prADH1-HA-Csg2-ΔTM9	This paper	HA-Csg2$^{\Delta TM9}$
p415-prADH1-85-GFP-86-Csg2	This paper	$^{85-}$GFP^{-86}-Csg2
p415-prADH1-Cps1-jGCamP7c	This paper	Cps1-jGCaMP7c
p415-prADH1-Sur2-GFP	This paper	Sur2-GFP
p425-prADH1-Aur1-HA	This paper	Aur1-HA
p415-prADH1-HA-Sur1	This paper	HA-Sur1
p415-prADH1-HA-Csh1	This paper	HA-Csh1
