# Supplementary material for: The ER calcium channel Csg2 integrates sphingolipid metabolism with autophagy
Source: Nat Commun. 2023 Jun 22;14:3725. doi: 10.1038/s41467-023-39482-6 (PMC10287731; doi:10.1038/s41467-023-39482-6)
Supplement: Supplementary file 3 — Description of Additional Supplementary Files [file 41467_2023_39482_MOESM3_ESM.pdf]

File name: Supplementary Data 1
Description: The transcriptome analysis of WT and csg2Δ yeast cells after starvation in SD-N medium. Experiments were set up in three biologically independent samples.

File name: Supplementary Data 2
Description: WT cells and csg2Δ cells were collected after 3 hours of starvation in SD-N medium and subject to lipidomics analysis. Experiments were set up in three biologically independent samples.

File name: Supplementary Data 3
Description: Q1 and Q3 masses and retention times of the indicated lipid species
