# Supplementary material for: The ER calcium channel Csg2 integrates sphingolipid metabolism with autophagy
Source: Nat Commun. 2023 Jun 22;14:3725. doi: 10.1038/s41467-023-39482-6 (PMC10287731; doi:10.1038/s41467-023-39482-6)
Supplement: Supplementary file 7 — Reporting Summary [file 41467_2023_39482_MOESM7_ESM.pdf]

nature portfolio

Corresponding author(s): Kefeng Lu

Last updated by author(s): Jun 1, 2023

Reporting Summary

Nature Portfolio wishes to improve the reproducibility of the work that we publish. This form provides structure for consistency and transparency in reporting. For further information on Nature Portfolio policies, see our Editorial Policies and the Editorial Policy Checklist.

Statistics

For all statistical analyses, confirm that the following items are present in the figure legend, table legend, main text, or Methods section.

n/a	Confirmed	
☐	☒	The exact sample size (*n*) for each experimental group/condition, given as a discrete number and unit of measurement
☐	☒	A statement on whether measurements were taken from distinct samples or whether the same sample was measured repeatedly
☐	☒	The statistical test(s) used AND whether they are one- or two-sided<br>*Only common tests should be described solely by name; describe more complex techniques in the Methods section.*
☒	☐	A description of all covariates tested
☐	☒	A description of any assumptions or corrections, such as tests of normality and adjustment for multiple comparisons
☐	☒	A full description of the statistical parameters including central tendency (e.g. means) or other basic estimates (e.g. regression coefficient) AND variation (e.g. standard deviation) or associated estimates of uncertainty (e.g. confidence intervals)
☐	☒	For null hypothesis testing, the test statistic (e.g. *F*, *t*, *r*) with confidence intervals, effect sizes, degrees of freedom and *P* value noted<br>*Give P values as exact values whenever suitable.*
☒	☐	For Bayesian analysis, information on the choice of priors and Markov chain Monte Carlo settings
☒	☐	For hierarchical and complex designs, identification of the appropriate level for tests and full reporting of outcomes
☒	☐	Estimates of effect sizes (e.g. Cohen's *d*, Pearson's *r*), indicating how they were calculated

Our web collection on statistics for biologists contains articles on many of the points above.

Software and code

Policy information about availability of computer code

Data collection	Western blotting images were taken by Image Lab (BioRad). Fluorescent images were collected by Zeiss observer 7 equipted with Apotome.
Data analysis	Fluorescent images were analyzed using Zeiss ZEN and Image J. Statistical analysis and figure were generated using GraphPad Prism 8.0.

For manuscripts utilizing custom algorithms or software that are central to the research but not yet described in published literature, software must be made available to editors and reviewers. We strongly encourage code deposition in a community repository (e.g. GitHub). See the Nature Portfolio guidelines for submitting code & software for further information.

Data

Policy information about availability of data

All manuscripts must include a data availability statement. This statement should provide the following information, where applicable:

- Accession codes, unique identifiers, or web links for publicly available datasets
- A description of any restrictions on data availability
- For clinical datasets or third party data, please ensure that the statement adheres to our policy

The data that support this study are available within the paper and in the Supplementary Information files and from the corresponding authors upon reasonable request. Source data are provided with this paper.

Human research participants

Policy information about studies involving human research participants and Sex and Gender in Research.

Reporting on sex and gender	n/a
Population characteristics	n/a
Recruitment	n/a
Ethics oversight	n/a

Note that full information on the approval of the study protocol must also be provided in the manuscript.

Field-specific reporting

Please select the one below that is the best fit for your research. If you are not sure, read the appropriate sections before making your selection.

☒ Life sciences ☐ Behavioural & social sciences ☐ Ecological, evolutionary & environmental sciences

For a reference copy of the document with all sections, see nature.com/documents/nr-reporting-summary-flat.pdf

Life sciences study design

All studies must disclose on these points even when the disclosure is negative.

Sample size	At least three independent replicates were used, with each containing sufficient cell amounts.
Data exclusions	No data were excluded.
Replication	Attempts at replication were successful.
Randomization	Experimental groups were chosen based on genotypes and treatments.
Blinding	Different genotypes were used alongside multiple controls.

Reporting for specific materials, systems and methods

We require information from authors about some types of materials, experimental systems and methods used in many studies. Here, indicate whether each material, system or method listed is relevant to your study. If you are not sure if a list item applies to your research, read the appropriate section before selecting a response.

Materials & experimental systems

n/a	Involved in the study	
☐	☒	Antibodies
☐	☒	Eukaryotic cell lines
☒	☐	Palaeontology and archaeology
☒	☐	Animals and other organisms
☒	☐	Clinical data
☒	☐	Dual use research of concern

Methods

n/a	Involved in the study	
☒	☐	ChIP-seq
☒	☐	Flow cytometry
☒	☐	MRI-based neuroimaging

Antibodies

Antibodies used	Mouse monoclonal antibodies to against HA-epitope (sc-7392; 1:2000 for IB) and GFP (sc-9996; 1:5000 for IB) were purchased from Santa Cruz Biotechnology, mouse monoclonal antibody to Pgk1(ab113687; clone 22C5D8; 1:10000 for IB) was purchased from Abcam. Mouse monoclonal antibody to Cherry (A2080; 1:5000 for WB) was purchased from Abbkine. Rabbit polyclonal antibody to Ape1 (1:1000 for IB) was a gift from Dr. Cong Yi (Zhejiang University). Rabbit monoclonal antibodies to p-Sch9 (Cat# 4858; 1:2000 for IB) and Sch9 (Cat# 2217; 1:1000 for IB) were purchased from Cell Signaling Technology. Anti-mouse antibodies conjugated to horseradish peroxidase (HRP) (D110087; 1:5000 for IB) and anti-rabbit antibodies conjugated to horseradish peroxidase (HRP) (D110058; 1:5000 for IB) were purchased from Sangon Biotech (Shanghai). Mouse monoclonal antibody to Cherry (A2080; 1:5000 for WB) was purchased from Abbkine.

Validation	Primary antibodies were validated by detecting no signal for negative controls and strong and specific signals for positive controls with appropriate size bands.

Eukaryotic cell lines

Policy information about cell lines and Sex and Gender in Research

Cell line source(s)	Sf9 cells were purchased from Invitrogen.
Authentication	The Sf9 cells were authenticated by the manufacturer.
Mycoplasma contamination	Negative.
Commonly misidentified lines (See ICLAC register)	*Name any commonly misidentified cell lines used in the study and provide a rationale for their use.*
